# A regulatory challenge for natural language processing (NLP)‐based tools such as ChatGPT to be legally used for healthcare decisions. Where are we now?

**DOI:** 10.1002/ctm2.1362

**Published:** 2023-08-07

**Authors:** Christian Baumgartner, Daniela Baumgartner

**Affiliations:** ^1^ Institute of Health Care Engineering with European Testing Center of Medical Devices Graz University of Technology Graz Austria; ^2^ Clinical Division of Pediatric Cardiology, Department of Pediatrics and Adolescent Medicine Medical University of Graz Graz Austria

**Keywords:** artificial intelligence, ChatGPT, healthcare decisions, natural language processing (NLP), regulatory approval, software as medical device (SaMD)

## 1 BACKGROUND

In the global debate about the use of Natural Language Processing (NLP)‐based tools such as ChatGPT in healthcare decisions, the question of their use as regulatory‐approved *Software as Medical Device (SaMD)* has not yet been sufficiently clarified. Currently, this discussion is conducted with an astonishing euphoria about countless clinical applications, including their opportunities, but also their pitfalls with potential errors in clinical use.^1^, ^2^, ^3^, ^4^, ^5^

Although the FDA and international regulatory authorities have already issued initial guideline documents for the development and approval of machine learning (ML)/artificial intelligence (AI)‐based tools as SaMD, a mandatory regulatory process for NLP‐based tools has not yet been fully clarified. ChatGPT therefore plays a special role in these considerations.

## 2 INTERNATIONAL LEGAL SITUATION FOR ML/AI‐BASED SAMD

In the United States, an FDA discussion paper from 2019 gives first ideas how to deliver safe and effective AI‐based software functionality for the total product lifecycle.^6^ A recent guidance document for clinical decision support software (September 2022) further clarifies the FDA's position on what qualifies as a regulable medical device, particularly with respect to AI‐driven clinical decision support tools.^7^ In March 2023, the FDA has published guidance on their algorithmic change control policy which discusses how it evaluates algorithms that are periodically updated, which is particularly relevant for NLP‐based tools such as ChatGPT.

In Europe, the medical device regulations (EU Regulations 2017/745 and 2017/746) are a major update to the way medical devices are regulated. According to the MDCG 2019‐11 guidance document, the gap in SaMD classification has been closed so far.^8^ A Product Watch Report of the European Commission from July 2020 provides an additional update on this topic, discussing AI, ML and statistical tools for risk estimation or decision support.^9^ The very recently published AI Act (June 2023), a proposed European law on artificial intelligence, will have far‐reaching consequences on medical device regulation in Europe in the near future.

The International Medical Device Regulators Forum (IMDRF)/Software as a Medical Device Working Group to harmonize the regulatory requirements published a possible risk categorization framework for SaMD in 2014, and a follow‐up document with more detailed information on ML/AI‐based software in 2022.^10^ The IMDRF and FDA recommendations allow for clearer identification of risk categories based on the *‘intended use’* for healthcare decisions in different medical situations or conditions (diagnosis, prognosis, prevention or treatment).

## 3 INITIAL REGULATORY SITUATION FOR NLP‐BASED TOOLS SUCH AS CHATGPT

**FIGURE 1:**
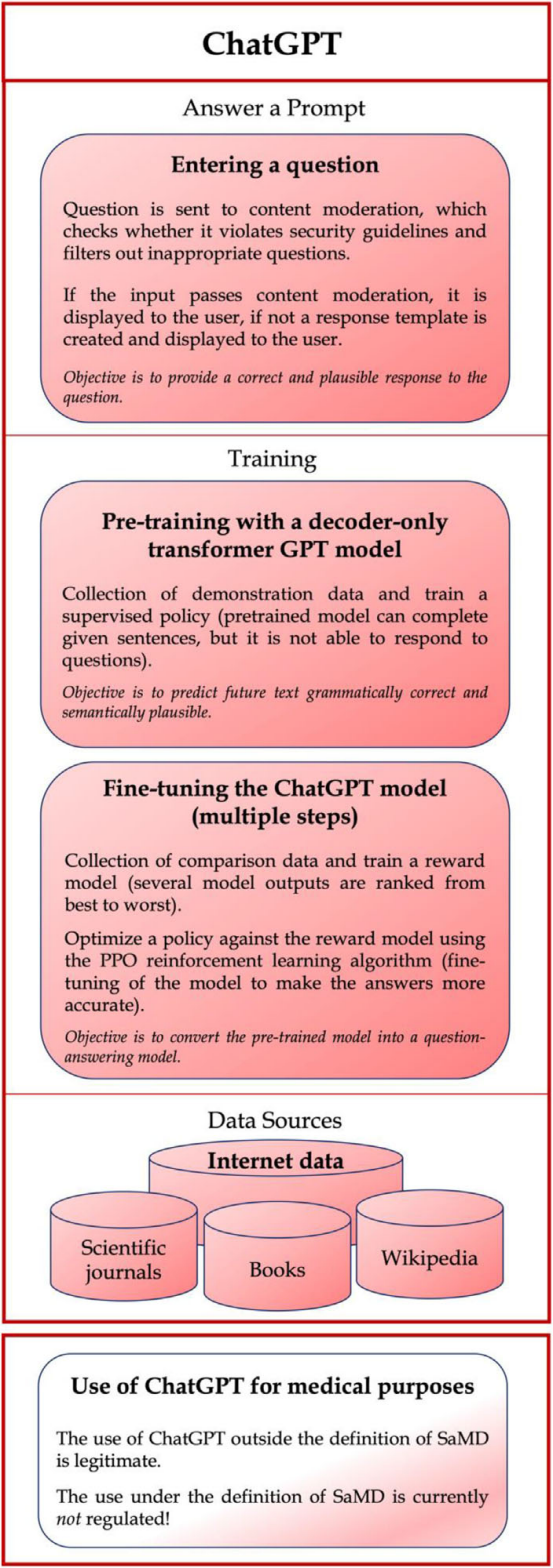
A simplified framework of ChatGPT, an AI‐based generative pre‐trained transformer (GPT) language model and its current situation for use. The ChatGPT framework diagram is based on information from the OpenAI website: https://openai.com/blog/chatgpt.

Currently, ChatGPT (Figure 1) is not intended by OpenAI for clinical use based on its terms of use (see https://openai.com/policies/terms‐of‐use), which clearly states that users should review its output for accuracy, that they make no warranties with respect to services, and that they have limited liability for any damages caused by its use. However, if the intended use of NLP‐based tools, beyond ChatGPT, for clinical purposes falls within the definition of an AI/ML‐based SaMD, regulatory approval is required.

In contrast to ‘locked’ software algorithms with fixed functions, for example, a classifier for clinical decision support, an ‘adaptive, continuous learning’ (non‐locked) algorithm changes its behavior. Because the standard medical device regulatory process is currently not designed for adaptive AI/ML technologies, additional efforts are needed by the regulators, although these algorithms have the potential to adapt and optimize software performance, in part in real‐time, to continuously improve patient health outcomes. Inherent changes to the algorithm are typically made and verified through a well‐defined and automated process to improve algorithm performance based on analysis and interpretation of new data.^6^ For clinical purposes, the data need to be evidence‐based and scientifically proven.

This is basically the concept of ChatGPT, but more complicated. ChatGPT is not actually an algorithm but merely refers to the user interface. GPT‐3.5‐turbo and GPT‐4 are underlying algorithms that drive ChatGPT. Whether the weights for GPT‐3.5‐turbo and GPT‐4 have not been updated since its release is not publicly known (no official confirmation by OpenAI). It can be speculated that the underlying language model has not been updated with new training data ( cf. the ChatGPT references back to September 2021), but the prompting, the structure of the model, and the chat component may have been updated. The fact that these models produce different output in response to the same prompt is actually due to its use of sampling in the output (see Figure 1). Especially this fact makes it difficult to simply modify ChatGPT (if intended by OpenAI) for regulatory approval as a SaMD in its current architecture. However, this need not apply to the architectures of other NLP‐based tools.

## 4 CLINICAL EXAMPLE AND REGULATORY CHALLENGES

The *intended use* of an NLP‐based SaMD to support clinical decisions, for example for the diagnosis and prognosis of the Marfan syndrome, a multisystemic disorder, could be as follows:

‘The NLP‐based software supports diagnostic and prognostic decision‐making in patients with suspected Marfan syndrome (ICD‐10 code: Q87.4), taking into account qualitative and quantitative information on the symptomatology of the cardiac, skeletal, ocular, skin, and pulmonary systems, as well as the genetic profile’.

### 4.1 A possible prompt according to the intended use

‘An 8‐years old patient carries an FBN1 mutation variant of c.3217G  >  T. Currently, only mild symptoms in the skeletal system are observed. Is aortic dilatation or dissection expected during the next 5 years?'

The problem with ChatGPT is that the model produces different output (variations) in response to the same prompt, so regulatory approval of medical software based on a ChatGPT architecture remains a challenge and will require major efforts to make such NLP‐tools available for clinical use. This behavior has significant implications for the relevant medical software certification requirements, including the IEC 62304 standard for the software life cycle processes, especially the development and maintenance process, usability engineering (IEC 62366), risk management (ISO 14971), the clinical evaluation to demonstrate clinical validity, among other requirements for which regulators are encouraged to define new or modified processes.

## 5 CONCLUSION

Beyond the euphoric discussion within the international medical community, there is a high urgency for a regulated use of NLP‐based tools to protect physicians and patients from potential errors and harms. All stakeholders—software developers, scientists, ethicists, healthcare professionals and healthcare providers, patient initiatives, regulators and governmental agencies—are called upon to raise public awareness of this important and urgent issue by taking the first binding steps.

## CONFLICT OF INTEREST STATEMENT

The authors declare that they have no conflict of interest.
